# Turkish Adaptation and Preliminary Psychometric Evaluation of the Mirror Image Comfort and Avoidance Scale (MICAS) in University Students

**DOI:** 10.3390/bs16101741

**Published:** 2026-09-24

**Authors:** Gamze Aktaş Kırat, Meserret Aslan, Fadime Bayrı Bingöl

**Affiliations:** 1 Department of Psychiatric Nursing, Faculty of Health Sciences, Marmara University, Maltepe, Istanbul 34854, Türkiye; gamze.aktas@fbu.edu.tr; 2 Department of Midwifery, Faculty of Health Sciences, Istanbul Atlas University, Istanbul 34408, Türkiye; 3 Department of Midwifery, Faculty of Health Sciences, Marmara University, Maltepe, Istanbul 34854, Türkiye; fadime.bingol@marmara.edu.tr

**Keywords:** body image, mirror viewing, psychometric evaluation, cultural adaptation, reliability, university students

## Abstract

Mirror viewing is closely related to body image and self-evaluation. The Mirror Image Comfort and Avoidance Scale (MICAS) assesses individuals’ comfort with and avoidance of viewing their own reflection. This study aimed to culturally adapt the MICAS into Turkish and evaluate its psychometric properties among university students. This methodological study was conducted with 407 university students between January and June 2025. Structural validity was examined using exploratory factor analysis (EFA) and confirmatory factor analysis (CFA), internal consistency was assessed using Cronbach’s alpha coefficients, and temporal stability was examined in a subgroup of 25 participants from the main sample who completed the scale twice approximately two weeks apart. EFA supported a 17-item, two-factor structure explaining 57.56% of the total variance. CFA using robust maximum likelihood estimation indicated an acceptable model fit (SB-χ^2^/df = 2.19, RMSEA = 0.054, SRMR = 0.068, CFI = 0.930, TLI = 0.919). Cronbach’s alpha coefficients were 0.83 for the Comfort subscale and 0.93 for the Avoidance subscale. Test–retest correlations were very strong for both subscales (r = 0.987–0.993, *p* < 0.001). The Turkish MICAS demonstrated an acceptable two-factor structure, good to excellent internal consistency, and strong test–retest associations, providing preliminary psychometric evidence for its use in research among university students. Further studies examining convergent, discriminant, criterion-related validity, and measurement invariance in more diverse populations are warranted.

## 1. Introduction

Body image is a multidimensional construct encompassing individuals’ perceptions, evaluations, thoughts, emotions, and behaviors related to their physical appearance. Rather than reflecting only satisfaction or dissatisfaction with appearance, contemporary conceptualizations emphasize positive body image, embodiment, acceptance, and the ways in which individuals interact with and experience their bodies (Piran et al., 2020; Tylka & Wood-Barcalow, 2015). Visual self-perception constitutes an important component of this experience, and mirrors represent one of the most common means through which individuals encounter and evaluate their own physical appearance. However, commonly used body image measures primarily assess broad constructs such as body satisfaction, body appreciation, appearance concerns, or body image flexibility, rather than individuals’ specific experiences while viewing themselves in a mirror. This creates a distinct measurement gap regarding mirror-viewing comfort and avoidance.

Mirror viewing is not simply a passive act of visual observation. Experimental and neurocognitive evidence suggests that viewing one’s own reflection contributes to self-recognition and body ownership and may influence emotional and cognitive responses to the body (Jenkinson & Preston, 2015; Preston et al., 2015). The Neurocognitive Mirror-Viewing Model further proposes that mirror exposure involves an interaction among visual self-recognition, memory processes, neurological responses, and autonomic reactions, particularly when the reflected body is associated with distressing or traumatic experiences (Freysteinson, 2020). Accordingly, mirror viewing may range from a routine and emotionally neutral activity to an experience characterized by discomfort, distress, or active avoidance.

The psychological effects of mirror exposure also appear to vary according to individuals’ existing body-related attitudes and the context in which mirror viewing occurs. Experimental research has shown that mirror gazing may increase self-focused attention and influence body-related evaluations, shame, and self-esteem (Barnier & Collison, 2019). Similarly, women exercising in mirrored environments have reported greater social physique anxiety and more frequent appearance comparisons than those exercising without mirrors (Frayeh & Lewis, 2018). Mirror exposure has also been discussed in relation to body dissatisfaction and appearance-focused attention, suggesting that the behavioral response to one’s reflection may provide information that is not fully captured by conventional measures of general body satisfaction (Mulkens & Jansen, 2009).

To address this measurement gap, Freysteinson et al. (2022) developed the Mirror Image Comfort and Avoidance Scale (MICAS) as a specific instrument for assessing the mirror-viewing experience. The final instrument consists of 17 items representing two related but conceptually distinct dimensions: Mirror Comfort and Mirror Avoidance. The original validation study demonstrated satisfactory factorial validity and high internal consistency for both subscales, with Cronbach’s alpha coefficients of 0.90 for Comfort and 0.94 for Avoidance (Freysteinson et al., 2022).

Subsequent research has extended the evaluation of the MICAS beyond the clinical and rehabilitation populations involved in its initial development. Validation in a general population demonstrated that Mirror Comfort and Mirror Avoidance were significantly associated with psychological variables including self-esteem, body appreciation, depression, anxiety, and stress, providing further evidence that mirror-viewing experiences are related to broader aspects of psychological well-being (Freysteinson et al., 2025). However, the present adaptation study did not include external measures of body image, self-esteem, or psychological distress; therefore, associations with these related constructs were not evaluated in the Turkish sample.

The availability of culturally appropriate measures is particularly important because body image and appearance-related behaviors are shaped by cultural norms, social expectations, interpersonal relationships, and culturally specific meanings attached to the body. The MICAS was originally developed and evaluated in a U.S.-based context, including adults recruited through amputation, veteran, and prosthesis-related organizations (Freysteinson et al., 2022). In contrast, Türkiye represents a cultural context in which Westernized appearance ideals coexist with more traditional and family-oriented social influences. Previous research among Turkish university students has shown that appearance- and eating-related attitudes are associated not only with individual factors but also with social influences such as parental traditionality and social comparison (Altuğ et al., 2000; Oksuz, 2008). Moreover, research on sociocultural attitudes toward appearance in Turkish college students has emphasized the simultaneous influence of Western and Eastern values and the roles of family, peers, media, and significant others in shaping appearance ideals (Cihan et al., 2016). These sociocultural differences may be particularly relevant to MICAS items referring to perceived attractiveness, other people viewing one’s reflection, intimacy, and viewing one’s naked reflection. Consequently, a direct linguistic translation may not be sufficient to ensure that such items carry equivalent meanings in the Turkish cultural context. Cross-cultural adaptation therefore requires systematic evaluation of linguistic, conceptual, and cultural equivalence, followed by empirical assessment of the psychometric properties of the adapted instrument (Hernández et al., 2020). In addition, rigorous scale validation requires examination of factor structure, item performance, internal consistency, and temporal stability to determine whether the proposed construct is measured reliably in the target population (Boateng et al., 2018).

This issue may be particularly relevant among university students. University years coincide with a developmental period in which appearance-related self-evaluation, social comparison, interpersonal relationships, and body image concerns may be especially salient. Mirror-viewing behaviors may therefore provide a distinct perspective on how university students experience and respond to their physical appearance. However, to date, no validated Turkish-language instrument has been available specifically to assess mirror-viewing comfort and avoidance.

Therefore, the present study aimed to translate and culturally adapt the MICAS into Turkish and to conduct a preliminary psychometric evaluation focusing on its structural validity, item performance, internal consistency, and temporal stability in a sample of university students. It was hypothesized that the Turkish version would retain the original two-factor structure comprising Mirror Comfort and Mirror Avoidance and demonstrate satisfactory internal consistency and temporal stability.

## 2. Materials and Methods

### 2.1. Study Design

This methodological study was conducted to perform the cross-cultural adaptation of the Mirror Image Comfort and Avoidance Scale (MICAS) into Turkish and to evaluate the psychometric properties of the Turkish version among university students. The study was conducted between January and June 2025. The psychometric evaluation was interpreted within Messick’s unified framework of validity, which conceptualizes validity as the degree to which empirical evidence and theoretical rationale support the interpretation and use of assessment scores rather than as an inherent property of an instrument itself (Messick, 1995). Within this framework, the present study focused primarily on evidence related to content and internal structure. Content-related evidence was addressed through translation, expert evaluation, and pilot testing, whereas evidence based on internal structure was examined through item analyses, exploratory factor analysis, and confirmatory factor analysis. Internal consistency and temporal stability were additionally examined to evaluate the reliability of the resulting scores. Evidence based on relationships with external variables, generalizability across populations, and measurement invariance was beyond the scope of the present preliminary evaluation and is identified as an area for future research.

The cross-cultural adaptation process included permission from the original scale developer, forward translation, synthesis of the translated versions, linguistic review, back-translation, expert evaluation, pilot testing, structural validity analyses, internal consistency assessment, and test–retest reliability assessment.

### 2.2. Participants and Sampling

The study population consisted of students enrolled at universities in Türkiye. Participants were recruited using convenience sampling. Study announcements containing brief information about the research and a link to the online questionnaire were disseminated through WhatsApp groups comprising university students that were accessible to the researchers through their academic roles and student networks. Because recruitment was conducted through student WhatsApp groups, the total number of individuals who viewed the study invitation could not be determined; therefore, a conventional response rate could not be calculated. The Google Forms survey was configured to allow only one response per account to reduce the possibility of duplicate submissions. Eligibility was assessed based on the inclusion criteria presented in the online survey. Only participants who met the eligibility criteria, provided electronic informed consent, and completed the required study forms were included in the final dataset. A total of 407 eligible participants with complete study data were included in the psychometric analyses.

During the initial planning of the study, a participant-to-item ratio of 10:1 was used as a pragmatic recruitment benchmark for the 17-item MICAS. Recruitment continued beyond this initial target, and a total of 407 eligible university students with complete data were ultimately included in the psychometric analyses, corresponding to approximately 24 participants per item. Although the eligibility criterion specified a minimum age of 18 years and no upper age limit was imposed, the observed age range in the final sample was 18–27 years. However, because both exploratory factor analysis (EFA) and confirmatory factor analysis (CFA) were conducted using the same full sample, the CFA findings should be interpreted as an internal evaluation of model fit rather than as an independent confirmation of the factor structure.

The inclusion criteria were as follows: (1) being 18 years of age or older, (2) being enrolled as a university student, (3) having no history of aesthetic/cosmetic surgery, and (4) having no self-reported psychiatric diagnosis. These criteria were applied to establish the preliminary psychometric properties of the Turkish MICAS in a relatively homogeneous, nonclinical student sample. Individuals with a self-reported psychiatric diagnosis were excluded to reduce clinical heterogeneity that could influence body-related self-evaluation and mirror-viewing experiences, whereas those with a history of aesthetic/cosmetic surgery were excluded because such procedures may substantially affect body perception and responses to one’s physical appearance. These exclusions were intended to reduce potential sources of heterogeneity during the initial adaptation of the instrument rather than to imply that the MICAS is unsuitable for these populations. Participants who did not complete the data collection forms or withdrew from the study were excluded.

### 2.3. Translation and Cross-Cultural Adaptation of the MICAS

Permission to translate and culturally adapt the MICAS into Turkish was obtained from the scale developer, Wyona M. Freysteinson, via e-mail before initiating the adaptation process.

The linguistic adaptation was conducted using a forward- and back-translation procedure. Forward translation refers to translating the original instrument from the source language into the target language, whereas back translation involves independently translating the reconciled target-language version back into the source language to evaluate its semantic and conceptual equivalence with the original instrument. First, the original English version of the MICAS was independently translated into Turkish by an English language lecturer and a clinical psychologist. One translator was informed about the purpose and conceptual background of the MICAS, whereas the other was not provided with detailed information about the instrument. The two forward translations were subsequently reviewed and compared by the research team. Differences in wording or meaning were discussed, and decisions were reached by consensus, taking into account semantic accuracy, conceptual equivalence, comprehensibility, and cultural appropriateness. Based on this process, a reconciled Turkish version was produced.

As an additional linguistic quality-control step, the reconciled Turkish version was reviewed by a Turkish literature teacher whose native language was Turkish. The revised Turkish version was then independently back translated into English by a clinical psychologist and an English language lecturer who had not previously seen the original MICAS. The back-translated versions were compared with the original instrument to identify potential discrepancies in meaning and to further assess semantic and conceptual equivalence.

Following the translation and linguistic review process, the preliminary Turkish version of the MICAS was evaluated by 9 experts with doctoral degrees and expertise in psychiatric nursing. Experts independently rated each item using a 4-point scale ranging from 1 (“requires major revision”) to 4 (“very appropriate”). The evaluation addressed linguistic accuracy, conceptual equivalence with the original item, cultural appropriateness, clarity, and the extent to which each item adequately represented the constructs of mirror-viewing comfort and avoidance. Ratings of 3 or 4 were considered indicative of content validity. The item-level content validity index (I-CVI) was calculated by dividing the number of experts who rated an item as 3 or 4 by the total number of experts. The scale-level content validity index using the average method (S-CVI/Ave) was calculated as the mean of the I-CVI values across all 17 items. An I-CVI of ≥0.78 and an S-CVI/Ave of ≥0.90 were considered indicative of satisfactory content validity (Polit et al., 2007). Items were reviewed and revised, where necessary, based on both the quantitative ratings and the experts’ qualitative feedback before pilot testing.

### 2.4. Pilot Testing

Following translation and expert evaluation, the preliminary Turkish version of the MICAS was pilot tested with 20 university students from the target population to evaluate item clarity, comprehensibility, and ease of response. Participants were specifically asked whether they experienced difficulty understanding or answering any item and were invited to comment on wording that they perceived as unclear, awkward, or culturally inappropriate. Feedback obtained during pilot testing was reviewed by the research team, and wording was revised where necessary before administration of the final Turkish version to the main study sample. Formal think-aloud interviews were not conducted; instead, the pilot testing was used as a respondent-based comprehension check to complement the expert review.

### 2.5. Data Collection

Data were collected electronically using a questionnaire created in Google Forms between January and June 2025. The survey link was distributed through WhatsApp groups comprising university students. Before accessing the study questionnaires, potential participants were presented with information regarding the purpose and procedures of the study, voluntary participation, confidentiality, and their right to withdraw from the study. Electronic informed consent was obtained before participants proceeded to the questionnaire.

The questionnaire required approximately 10–15 min to complete and consisted of the Demographic Information Form and the Turkish version of the Mirror Image Comfort and Avoidance Scale.

### 2.6. Data Collection Instruments

#### 2.6.1. Demographic Information Form

The Demographic Information Form was developed by the researchers based on the relevant literature. It included questions regarding age, height, weight, academic department or program, marital status, employment status, perceived economic status, smoking and alcohol use, regular exercise, chronic and physical illnesses, and participants’ general satisfaction with their bodies.

#### 2.6.2. Mirror Image Comfort and Avoidance Scale

The Mirror Image Comfort and Avoidance Scale (MICAS) was developed by Freysteinson et al. to assess individuals’ experiences of comfort with and avoidance of viewing their own reflection in a mirror (Freysteinson et al., 2022). The MICAS was designed as a measure specifically addressing the mirror-viewing experience and consists of 17 items distributed across two subscales: Mirror Comfort (9 items) and Mirror Avoidance (8 items).

Each item is rated on a five-point Likert-type scale, ranging from 1 (“strongly disagree”) to 5 (“strongly agree”). In the original MICAS, two negatively worded items belonging to the Comfort subscale are reverse scored. Higher scores on the Comfort subscale indicate greater comfort when viewing oneself in a mirror, whereas higher scores on the Avoidance subscale indicate greater mirror avoidance. The possible score ranges are 9–45 for the Comfort subscale and 8–40 for the Avoidance subscale.

In the original validation study, the MICAS demonstrated a two-factor structure. Internal consistency coefficients were α = 0.90 for the Comfort subscale and α = 0.94 for the Avoidance subscale. The original study also provided evidence for factorial validity, construct validity, and temporal stability (Freysteinson et al., 2022).

For the Turkish adaptation, the items were organized according to their respective subscales. The Comfort subscale comprised Items 1–9, and the Avoidance subscale comprised Items 10–17. Because the two negatively worded Comfort items in the original instrument correspond to Items 5 and 7 in the reordered Turkish version, these two items were reverse scored when calculating the Comfort subscale score. To facilitate comparison with the original MICAS, the correspondence between the original item numbers and the reordered Turkish item numbers is presented in Table 1.

### 2.7. Test–Retest Procedure

Temporal stability was assessed in a subgroup of participants from the main psychometric sample. Approximately 15 days after the initial administration, a retest invitation was shared through the same student WhatsApp groups used for the initial data collection, inviting students who had previously completed the survey to participate in the second administration. Twenty-five participants from the original sample voluntarily completed the Turkish MICAS a second time. Participants’ first- and second-administration responses were matched at the individual level, and the paired data were used for the test–retest analysis.

### 2.8. Statistical Analysis

Statistical analyses were performed using IBM SPSS Statistics version 29.0 and LISREL 8.51. A two-sided *p* value of <0.05 was considered statistically significant. Descriptive statistics were used to summarize participant characteristics and MICAS scores. Demographic variables measured on a continuous scale, such as age, height, weight, and body mass index, as well as subscale scores, were summarized using means and standard deviations, whereas categorical variables were summarized using frequencies and percentages. Individual MICAS items were considered ordinal and were additionally described using medians, skewness, kurtosis, and floor and ceiling response proportions.

#### 2.8.1. Item Analysis and Internal Consistency

Item performance was evaluated using Spearman rank-order correlations because the MICAS items were measured using a five-point ordinal response format. Corrected item–total correlations were calculated within each subscale by correlating each item with the corresponding subscale score after excluding that item from the subscale total.

Item-level distributions were additionally examined using means, standard deviations, medians, skewness, kurtosis, floor and ceiling response proportions, and missing-data rates. Floor and ceiling proportions were defined as the percentages of participants selecting the lowest (1) and highest (5) response categories, respectively. Descriptive item statistics were calculated using the original response coding prior to reverse scoring. Item discrimination was additionally examined by comparing scores between the upper and lower 27% groups.

Internal consistency was assessed using Cronbach’s alpha coefficients separately for the Comfort and Avoidance subscales. Cronbach’s alpha coefficients of ≥0.70 were considered indicative of acceptable internal consistency.

#### 2.8.2. Structural Validity

Prior to the exploratory factor analysis (EFA), the suitability of the data for factor analysis was evaluated using the Kaiser–Meyer–Olkin (KMO) measure of sampling adequacy and Bartlett’s test of sphericity. EFA was conducted using principal components extraction with Direct Oblimin rotation, as the Comfort and Avoidance dimensions were theoretically expected to be related. The number of factors was determined considering eigenvalues greater than 1 and the conceptual structure of the original MICAS. Factor loadings of ≥0.40 were considered salient for interpretation, while secondary loadings of ≥0.30 were examined as potential cross-loadings. Communalities and the correlation between the rotated factors were also examined.

The total sample was not split into separate EFA and CFA subsamples. The two-factor structure identified through EFA was subsequently evaluated using confirmatory factor analysis (CFA) in LISREL 8.51 using the same full sample. Accordingly, the CFA was regarded as an internal model-fit evaluation rather than an independent cross-validation of the factor structure. Prior to CFA, multivariate normality was assessed using Mardia’s multivariate skewness and kurtosis coefficients (Mardia, 1970). Because the data showed evidence of multivariate non-normality, the CFA model was estimated using robust maximum likelihood estimation with the Satorra–Bentler correction (Satorra & Bentler, 1994). The five-point Likert-type items were treated as approximately continuous indicators, while the robust correction was used to account for departures from multivariate normality. Model fit was evaluated using the Satorra–Bentler scaled chi-square statistic (SB-χ^2^), degrees of freedom (df), RMSEA, SRMR, CFI, and TLI.

#### 2.8.3. Temporal Stability

Paired-samples t-tests were used to compare mean Comfort and Avoidance subscale scores between the first and second administrations. Pearson correlation coefficients were used to examine the associations between scores obtained at the two measurement occasions. To examine potential differences associated with participation in the retest, the retest subgroup (*n* = 25) was compared with participants who did not complete the retest (*n* = 382) in terms of age, BMI, and sex. Age and BMI were compared using independent-samples t-tests, whereas sex distribution was compared using Fisher’s exact test. Statistical significance was set at *p* < 0.05.

### 2.9. Ethical Considerations

The study was approved by the Marmara University Faculty of Health Sciences Non-Interventional Clinical Research Ethics Committee (26 December 2024; Approval No. 163).

The study was conducted in accordance with the principles of the Declaration of Helsinki. Participation was voluntary. All participants received information about the purpose and procedures of the study, confidentiality of the data, and their right to withdraw without any consequences. Electronic informed consent was obtained from all participants before completion of the online questionnaire.

## 3. Results

A total of 407 university students were included in the final psychometric analyses of the Turkish version of the MICAS.

The mean age of the participants was 19.78 ± 1.53 years (range: 18–27), and 79.9% were female. The mean body mass index (BMI) was 23.02 ± 3.52 kg/m^2^. Most participants were nursing students (54.1%), single (97.3%), and reported a moderate economic status (73.2%). Detailed sociodemographic and health-related characteristics of the participants are presented in Table 2.

### 3.1. Content Validity

Expert evaluation of the preliminary Turkish MICAS indicated strong scale-level content validity. Across the 17 items, I-CVI values ranged from 0.67 to 1.00. Fourteen items had I-CVI values between 0.89 and 1.00, Item 6 had an I-CVI of 0.78, and Items 3 and 16 had I-CVI values of 0.67. The S-CVI/Ave was 0.93, exceeding the recommended scale-level criterion of 0.90. Items 3 and 16, which showed lower expert agreement, were retained for further evaluation during pilot testing and the subsequent psychometric analyses.

### 3.2. Item-Level Descriptive Statistics

Item-level descriptive statistics are presented in Table 3. No missing responses were observed for any of the 17 MICAS items. Item means ranged from 1.36 to 3.73. The Comfort items generally showed relatively balanced distributions, although Items 5 and 7 showed higher proportions of responses at the lowest category. In contrast, the Avoidance items showed pronounced floor effects, with 49.4–73.5% of participants selecting the lowest response category for Items 10–17. Skewness and kurtosis were also greater for several Avoidance items, particularly Items 15–17, indicating non-normal and floor-concentrated item distributions.

### 3.3. Item Analysis and Internal Consistency Reliability

Corrected item–total correlations based on Spearman’s rank-order coefficient were statistically significant for all items (*p* < 0.001). For the Comfort subscale, corrected item–total correlations ranged from r_s_ = 0.265 to 0.750, whereas those for the Avoidance subscale ranged from r_s_ = 0.637 to 0.819. Item 8 showed the lowest corrected item–total correlation (r_s_ = 0.265), although it remained statistically significant and significantly discriminated between the lower and upper 27% groups (t = 6.08, *p* < 0.001) (Table 4). All items significantly differentiated between the upper and lower 27% groups. Cronbach’s alpha coefficients were 0.83 for the Comfort subscale and 0.93 for the Avoidance subscale.

### 3.4. Test–Retest Associations

Temporal stability was examined in a subgroup of 25 participants from the main psychometric sample who voluntarily completed the MICAS at both measurement occasions. Their first-administration responses were included in the main psychometric sample of 407 participants, whereas their second-administration responses were used only for the test–retest analysis. The retest subgroup did not differ significantly from participants who did not complete the retest in terms of age (19.68 ± 1.38 vs. 19.79 ± 1.54 years, *p* = 0.709), BMI (22.70 ± 3.42 vs. 23.04 ± 3.54 kg/m^2^, *p* = 0.630), or sex distribution (*p* = 0.194). However, all participants in the retest subgroup were enrolled in nursing or midwifery programs, whereas the remainder of the main sample also included students from other academic programs. No statistically significant differences were observed between the first and second administrations for the Comfort subscale (32.28 ± 5.73 vs. 32.40 ± 5.74; t = −0.901, *p* = 0.376) or the Avoidance subscale (10.08 ± 2.77 vs. 9.92 ± 2.56; t = 1.693, *p* = 0.103). Very strong positive associations were observed between the two administrations, with Pearson correlation coefficients of r = 0.993 (*p* < 0.001) for the Comfort subscale and r = 0.987 (*p* < 0.001) for the Avoidance subscale (Table 5). These findings indicate strong associations between scores obtained at the two measurement occasions; however, Pearson correlations reflect association rather than absolute agreement.

### 3.5. Exploratory Factor Analysis

The data were suitable for factor analysis, as indicated by a Kaiser–Meyer–Olkin (KMO) value of 0.923 and a statistically significant Bartlett’s test of sphericity, χ^2^(136) = 4020.36, *p* < 0.001. Principal components extraction with Direct Oblimin rotation supported a two-factor structure corresponding to Mirror Comfort and Mirror Avoidance. The first two components had eigenvalues of 7.374 and 2.410 and accounted for 43.38% and 14.18% of the variance, respectively, explaining 57.56% of the total variance. The primary pattern coefficients ranged from 0.430 to 0.811 for the Comfort items and from 0.721 to 0.856 for the Avoidance items. Communalities ranged from 0.181 to 0.762, corresponding to uniqueness values ranging from 0.238 to 0.819. Item 5 showed a secondary loading of −0.303 on the Avoidance factor in addition to its primary loading of 0.430 on the Comfort factor, whereas Item 15 showed a secondary loading of 0.303 on the Comfort factor in addition to its primary loading of 0.830 on the Avoidance factor. The remaining items did not demonstrate secondary loadings ≥ 0.30. The two factors were moderately and negatively correlated (r = −0.435), indicating that greater mirror comfort was associated with lower mirror avoidance while supporting their treatment as related but distinct dimensions (Table 6).

### 3.6. Confirmatory Factor Analysis

Prior to CFA, multivariate normality was evaluated using Mardia’s multivariate skewness and kurtosis coefficients. Mardia’s multivariate skewness was 116.62, χ^2^(969) = 7910.94, *p* < 0.001, and multivariate kurtosis was 584.22, z = 103.67, *p* < 0.001, indicating a significant departure from multivariate normality. Accordingly, the two-factor model was estimated using robust maximum likelihood estimation with the Satorra–Bentler correction. The model demonstrated an acceptable fit to the data: SB-χ^2^(118) = 257.91, *p* < 0.001, SB-χ^2^/df = 2.19, RMSEA = 0.054 (90% CI [0.045, 0.063]), SRMR = 0.068, CFI = 0.930, and TLI = 0.919. No correlated residuals or post hoc model modifications were specified. The standardized latent correlation between Mirror Comfort and Mirror Avoidance was moderate and negative (φ = −0.57), indicating that the two dimensions were related but not redundant. Because the CFA was conducted using the same sample as the EFA, these findings should be interpreted as an internal evaluation of model fit rather than as an independent confirmation of the factor structure (Table 7).

## 4. Discussion

The present study aimed to culturally adapt the Mirror Image Comfort and Avoidance Scale (MICAS) into Turkish and conduct a preliminary psychometric evaluation among university students. Overall, the findings provided preliminary evidence supporting the two-dimensional structure of the Turkish MICAS, comprising Mirror Comfort and Mirror Avoidance. The Turkish version demonstrated good to excellent internal consistency, adequate item discrimination, and strong test–retest associations.

Expert evaluation also provided quantitative evidence regarding content validity. The S-CVI/Ave was 0.93, indicating strong scale-level content validity. Most items demonstrated high expert agreement; however, Items 3 and 16 each had an I-CVI of 0.67. Despite this lower initial expert agreement, both items showed strong empirical performance in the main sample, with corrected item–total correlations of r_s_ = 0.691 and r_s_ = 0.730 and primary factor loadings of 0.803 and 0.841, respectively. These findings supported retention of both items in the Turkish version, although future cognitive interviewing may help further clarify their cultural interpretation.

The two-factor structure identified in the present study is consistent with the conceptual structure of the original MICAS developed by Freysteinson et al. (2022). In the original instrument, Mirror Comfort and Mirror Avoidance emerged as related but distinct dimensions of the mirror-viewing experience. Consistent with this conceptualization, the latent correlation between the two factors in the present study was moderate and negative (φ = −0.57), suggesting that greater comfort with one’s mirror image was associated with lower avoidance while also indicating that the two dimensions were not redundant. Similarly, the exploratory factor analysis in the present study identified two factors comprising the same 17 items, with Items 1–9 representing Mirror Comfort and Items 10–17 representing Mirror Avoidance. Together, these factors explained 57.56% of the total variance. Primary pattern coefficients ranged from 0.430 to 0.811 for the Comfort items and from 0.721 to 0.856 for the Avoidance items. The preservation of the original two-dimensional conceptual structure following Turkish cultural adaptation provides evidence supporting the structural validity of the adapted instrument.

The confirmatory factor analysis provided additional support for the two-factor structure. Because multivariate normality was significantly violated, the model was estimated using robust maximum likelihood estimation with the Satorra–Bentler correction. The robust model yielded SB-χ^2^(118) = 257.91, *p* < 0.001, SB-χ^2^/df = 2.19, RMSEA = 0.054 (90% CI [0.045, 0.063]), SRMR = 0.068, CFI = 0.930, and TLI = 0.919. Taken together, these indices indicated an acceptable representation of the observed data. In addition, the moderate negative latent correlation between Mirror Comfort and Mirror Avoidance (φ = −0.57) supported the interpretation of these dimensions as related but distinct constructs. However, because the EFA and CFA were conducted using the same sample, the CFA findings should be interpreted as an internal evaluation of model fit rather than as independent confirmation of the factor structure. Overall, the findings are broadly consistent with the original development study, in which the 17-item MICAS also demonstrated a two-factor structure comprising Mirror Comfort and Mirror Avoidance (Freysteinson et al., 2022).

The internal consistency findings also supported the reliability of the Turkish MICAS. Cronbach’s alpha coefficients were 0.83 for the Comfort subscale and 0.93 for the Avoidance subscale. These values indicate good and excellent internal consistency, respectively. The results are comparable with those reported in the original development study, in which Cronbach’s alpha coefficients were 0.90 for Mirror Comfort and 0.94 for Mirror Avoidance (Freysteinson et al., 2022). More recently, validation of the MICAS in a general adult population yielded alpha coefficients of 0.90 and 0.92 for the Comfort and Avoidance subscales, respectively (Freysteinson et al., 2025). Taken together, the consistency of reliability coefficients across populations supports the internal coherence of both MICAS dimensions, although the somewhat lower alpha coefficient observed for Comfort in the present sample may reflect cultural or sample-specific differences in the way certain aspects of mirror comfort are experienced.

The item analyses generally supported the performance of the individual items. Corrected item–total correlations based on Spearman’s rank-order coefficient were statistically significant for all items, and all items significantly discriminated between the lower and upper 27% groups (all *p* < 0.001). Item 8, however, showed comparatively weaker psychometric performance than the other Comfort items. Its corrected item–total correlation was relatively low (r_s_ = 0.265), and although its primary factor loading remained acceptable (0.471), its communality was low (h^2^ = 0.181), indicating that only a limited proportion of its variance was explained by the two-factor solution. Nevertheless, the item significantly discriminated between the lower and upper 27% groups and was retained because it remained conceptually relevant to the Mirror Comfort construct and corresponded to an item in the original MICAS. The comparatively weaker performance of this item may reflect its specific reference to a spouse or significant other viewing the respondent’s naked image in a bathroom mirror. Responses may therefore be influenced not only by mirror comfort but also by relationship status, intimacy, privacy norms, and sociocultural attitudes toward nudity. Future studies should examine the functioning of this item across different relationship statuses, genders, age groups, and cultural contexts before any modification or removal is considered.

The test–retest findings also supported the reproducibility of MICAS scores over the approximately 15-day interval. Very strong correlations were observed between the two administrations (r = 0.993 for Comfort and r = 0.987 for Avoidance). These values were higher than those reported in the original MICAS study, in which test–retest correlations of 0.81 for Comfort and 0.94 for Avoidance were observed over a two-week interval (Freysteinson et al., 2022). Nevertheless, Pearson correlations primarily reflect the association between repeated measurements rather than absolute agreement. Intraclass correlation coefficients are generally preferable when assessing test–retest agreement (Polit, 2014). Therefore, future studies should evaluate the temporal reliability of the Turkish MICAS using ICCs with 95% confidence intervals in larger retest samples.

The relevance of assessing mirror-viewing experiences extends beyond traditional measures of general body satisfaction. The MICAS specifically evaluates how individuals experience their own reflections, distinguishing comfort with mirror viewing from behavioral and emotional avoidance of mirrors. Recent evidence continues to demonstrate that mirror viewing can influence appearance-related self-evaluation. Experimental research among university students has shown that heightened self-focused attention during mirror gazing can adversely influence appearance evaluations and appearance-related experiences (Chuah & Suendermann, 2024). Accordingly, a culturally appropriate measure of both comfort and avoidance may be useful for investigating individual differences in responses to mirror exposure and their relationship with broader aspects of body image.

The use of the MICAS may be particularly relevant among university students. Emerging adulthood is a period in which appearance-related social comparison, self-evaluation, and body image concerns can be prominent, particularly among women. The availability of a Turkish measure that focuses specifically on the mirror-viewing experience may therefore facilitate research examining the relationships between mirror comfort and avoidance and constructs such as body appreciation, body dissatisfaction, self-esteem, anxiety, depression, social appearance anxiety, and appearance-related social comparison. In the general-population validation of the MICAS, mirror comfort and avoidance were significantly associated with anxiety, depression, stress, self-esteem, and body appreciation (Freysteinson et al., 2025). Future studies of the Turkish version should examine similar relationships to provide additional evidence for convergent and discriminant validity.

Several limitations should be considered when interpreting the findings. First, the study included university students recruited using convenience sampling; therefore, the findings may not be generalizable to non-student populations, older adults, or clinical populations. The exclusion of individuals with self-reported psychiatric diagnoses or a history of aesthetic/cosmetic surgery further limits generalizability to groups in whom mirror-related discomfort and avoidance may be particularly relevant. Second, EFA and CFA were conducted using the same sample. Therefore, the CFA findings represent an internal model-fit assessment rather than an independent cross-validation, and the two-factor structure should be replicated in an independent Turkish sample. Robust maximum likelihood estimation with the Satorra–Bentler correction was used to account for the observed violation of multivariate normality. Nevertheless, given the ordinal response format and pronounced floor effects observed for several items, future studies may further examine the factor structure using ordinal-data estimators based on polychoric correlations in independent samples. Third, participants were recruited through student WhatsApp groups using convenience sampling and completed an online self-report survey. This recruitment strategy may have introduced self-selection and network-related sampling biases, as students who were more closely connected to the academic and student networks through which the study was disseminated may have been more likely to encounter and participate in the study. Consequently, the sample may not fully represent the broader university student population in Türkiye. Fourth, the test–retest assessment was based on a relatively small subgroup of 25 participants from the main psychometric sample who voluntarily participated in the second administration. Because participation in the retest was voluntary, self-selection bias cannot be excluded, and the very strong test–retest correlations should therefore be interpreted cautiously. Although the retest subgroup did not differ from non-retest participants in age, BMI, or sex distribution, its restriction to nursing and midwifery students may further limit the representativeness of the temporal-stability findings. Moreover, temporal stability was assessed using Pearson correlation coefficients, which quantify the association between repeated measurements rather than absolute agreement. Future studies should therefore examine test–retest reliability in larger samples using intraclass correlation coefficients with 95% confidence intervals. Fifth, the present study did not include established external measures of body image, body appreciation, self-esteem, psychological distress, or related constructs; consequently, convergent, discriminant, and criterion-related validity could not be evaluated. Finally, measurement invariance across sex and other relevant demographic or clinical groups was not examined. Future studies should evaluate measurement invariance and replicate the psychometric properties of the Turkish MICAS across more diverse populations.

Despite these limitations, this study provides initial evidence supporting the cross-cultural applicability of the two-dimensional MICAS structure in Turkish university students. The preservation of the original Comfort and Avoidance dimensions, acceptable CFA fit, satisfactory internal consistency, and temporal stability support the use of the Turkish MICAS as a measure of mirror-viewing experiences in research involving this population.

## 5. Conclusions

The Turkish version of the MICAS demonstrated an acceptable two-factor structure comprising Mirror Comfort and Mirror Avoidance, together with good to excellent internal consistency and strong test–retest associations among university students. These findings provide preliminary psychometric evidence supporting its use for assessing mirror-viewing comfort and avoidance in research. Further studies using independent samples, robust estimators for ordinal data, external validation measures, and measurement invariance analyses are warranted.

## Figures and Tables

**Table 1:** Crosswalk Between the Original MICAS and the Reordered Turkish Version.

Turkish Item	Original MICAS Item	Subscale	Reverse Scored
1	2	Comfort	No
2	6	Comfort	No
3	7	Comfort	No
4	9	Comfort	No
5	10	Comfort	Yes
6	11	Comfort	No
7	14	Comfort	Yes
8	15	Comfort	No
9	16	Comfort	No
10	1	Avoidance	No
11	3	Avoidance	No
12	4	Avoidance	No
13	5	Avoidance	No
14	8	Avoidance	No
15	12	Avoidance	No
16	13	Avoidance	No
17	17	Avoidance	No

Note. Items were reordered in the Turkish version to group the Comfort and Avoidance items by subscale. Original MICAS Items 10 and 14 correspond to Turkish Items 5 and 7, respectively, and are reverse scored when calculating the Comfort subscale score.

**Table 2:** Sociodemographic and Health-Related Characteristics of the Participants (*n* = 407).

Characteristic	Category/Statistic	*n* (%) or Mean ± SD
**Age, years**	Mean ± SD (range)	19.78 ± 1.53 (18–27)
**Sex**	Female	325 (79.9)
	Male	82 (20.1)
**Height, cm**	Mean ± SD	167.04 ± 8.25
**Weight, kg**	Mean ± SD	64.41 ± 11.97
**BMI, kg/m^2^**	Mean ± SD	23.02 ± 3.52
**Department/program**	Nursing	220 (54.1)
	Midwifery	107 (26.3)
	Dialysis	40 (9.8)
	Anesthesia	18 (4.4)
	Medical Laboratory Techniques	12 (2.9)
	Operating Room Services	9 (2.2)
	Radiology	1 (0.2)
**Marital status**	Single	396 (97.3)
	Married	11 (2.7)
**Perceived economic status**	Good	49 (12.0)
	Moderate	298 (73.2)
	Poor	60 (14.7)
**Smoking**	Yes	71 (17.4)
	No	291 (71.5)
	Occasionally	41 (10.1)
	Quit	4 (1.0)
**Alcohol use**	Yes	32 (7.9)
	No	314 (77.1)
	Quit	61 (15)
**Exercise**	Yes	77 (18.9)
	No	167 (41)
	Occasionally	163 (40)
**Chronic illness**	Yes	38 (9.3)
	No	369 (90.7)
**General body satisfaction**	Yes	300 (73.7)
	No	107 (26.3)

**Table 3:** Item-Level Descriptive Statistics of the Turkish MICAS (*n* = 407).

Item	Mean	SD	Median	Skewness	Kurtosis	Floor, %	Ceiling, %	Missing, %
**Item 1**	3.67	0.93	4	−0.73	0.65	3.2	16.0	0.0
**Item 2**	3.73	0.96	4	−0.76	0.47	2.9	19.7	0.0
**Item 3**	3.63	1.03	4	−0.62	−0.04	3.9	19.9	0.0
**Item 4**	3.67	1.03	4	−0.84	0.39	4.9	19.2	0.0
**Item 5**	2.32	1.13	2	0.70	−0.20	26.3	5.7	0.0
**Item 6**	3.20	1.18	3	−0.31	−0.78	10.6	13.0	0.0
**Item 7**	2.51	1.23	2	0.45	−0.84	24.1	7.1	0.0
**Item 8**	2.95	1.22	3	−0.05	−0.77	16.7	12.3	0.0
**Item 9**	3.51	1.04	4	−0.40	−0.33	3.9	17.4	0.0
**Item 10**	1.71	0.87	2	1.37	2.14	49.4	1.7	0.0
**Item 11**	1.68	0.85	1	1.33	1.77	51.1	1.0	0.0
**Item 12**	1.59	0.87	1	1.79	3.37	59.0	1.7	0.0
**Item 13**	1.52	0.79	1	1.90	4.34	61.7	1.2	0.0
**Item 14**	1.59	0.87	1	1.68	2.77	59.2	1.2	0.0
**Item 15**	1.36	0.70	1	2.53	7.80	73.5	1.0	0.0
**Item 16**	1.47	0.79	1	2.02	4.70	65.8	1.2	0.0
**Item 17**	1.39	0.72	1	2.02	3.91	72.7	0.2	0.0

Note. Floor and ceiling values represent the percentages of participants selecting response categories 1 and 5, respectively. Descriptive statistics are based on the original item-response coding before reverse scoring. Items 5 and 7 are reverse scored when calculating the Comfort subscale score.

**Table 4:** Corrected Item–Total Correlations, Upper–Lower 27% Group Comparisons, and Internal Consistency of the Turkish MICAS (*n* = 407).

Turkish Item	Item Wording	Corrected Item–Total r_s_	t (Lower vs. Upper 27%)	*p*
**1**	I like what I see when I look in a mirror.	0.700	15.82	<0.001
**2**	I am comfortable looking in mirrors.	0.750	17.06	<0.001
**3**	Looking in the mirror helps me accept my body.	0.691	16.04	<0.001
**4**	The more I look in the mirror, the more I accept my body and the changes I am going through.	0.513	10.88	<0.001
**5 ^†^**	When looking in the mirror, I believe no one will ever find me attractive.	0.500	15.02	<0.001
**6**	I am okay with others looking at my reflection in a mirror.	0.454	11.24	<0.001
**7 ^†^**	When I look in the mirror, I desperately wish I could change my appearance.	0.489	14.78	<0.001
**8**	I am okay with my spouse/significant other looking at my naked image in the bathroom mirror.	0.265	6.08	<0.001
**9**	I always feel comfortable looking in mirrors.	0.562	13.18	<0.001
**10**	I hate mirrors.	0.705	14.63	<0.001
**11**	I avoid looking into reflective surfaces (e.g., windows or shiny surfaces).	0.819	16.39	<0.001
**12**	When I look in a mirror, I feel revulsion.	0.804	14.90	<0.001
**13**	Looking in the mirror is so difficult that I think I need to talk to someone.	0.767	12.87	<0.001
**14**	I am afraid to look in mirrors.	0.800	13.48	<0.001
**15**	I do not look in mirrors, not even to comb my hair or brush my teeth.	0.637	7.97	<0.001
**16**	I avoid the mirror because I am afraid of what I will see.	0.730	11.52	<0.001
**17**	I do not look in the mirror anymore.	0.710	10.35	<0.001

Note. r_s_ = Spearman’s rank-order correlation coefficient. Corrected item–total correlations were calculated within each respective subscale after excluding the target item from the subscale score. ^†^ Reverse-scored item. Cronbach’s α = 0.83 for Mirror Comfort and 0.93 for Mirror Avoidance.

**Table 5:** Test–Retest Associations of the Turkish MICAS (*n* = 25).

Scale/Subscale	First Assessment Mean ± SD	Second Assessment Mean ± SD	t	*p*	r	*p*
Comfort	32.28 ± 5.73	32.40 ± 5.74	−0.901	0.376	0.993	<0.001
Avoidance	10.08 ± 2.77	9.92 ± 2.56	1.693	0.103	0.987	<0.001

Note. r = Pearson correlation coefficient. Correlations were calculated to examine the association between scores obtained at the two measurement occasions.

**Table 6:** Exploratory Factor Analysis of the Turkish MICAS: Factor Loadings, Communalities, and Uniqueness.

Turkish Item	Comfort	Avoidance	h^2^	u^2^
Item 1	0.808	−0.048	0.689	0.311
Item 2	0.811	−0.086	0.726	0.274
Item 3	0.803	−0.009	0.651	0.349
Item 4	0.699	0.046	0.463	0.537
Item 5	0.430	−0.303	0.390	0.610
Item 6	0.608	0.037	0.352	0.648
Item 7	0.458	−0.201	0.330	0.670
Item 8	0.471	0.168	0.181	0.819
Item 9	0.690	−0.002	0.478	0.522
Item 10	0.179	0.721	0.664	0.336
Item 11	0.151	0.764	0.707	0.293
Item 12	0.141	0.763	0.695	0.305
Item 13	0.019	0.830	0.703	0.297
Item 14	0.038	0.856	0.762	0.238
Item 15	0.303	0.830	0.561	0.439
Item 16	0.027	0.841	0.728	0.272
Item 17	−0.039	0.856	0.706	0.294

Note. Pattern coefficients from principal components extraction with Direct Oblimin rotation are presented. Bold values indicate the primary loading of each item. h^2^ = communality; u^2^ = uniqueness (1 − h^2^). The correlation between the Comfort and Avoidance factors was −0.435. Eigenvalues for the two retained factors were 7.374 and 2.410, accounting for 57.56% of the total variance.

**Table 7:** Robust Confirmatory Factor Analysis Fit Indices for the Two-Factor Model of the Turkish MICAS (*N* = 407).

Fit Index	Observed Value
SB-χ^2^	257.91
df	118
*p*	<0.001
SB-χ^2^/df	2.19
RMSEA	0.054
RMSEA 90% CI	[0.045, 0.063]
SRMR	0.068
CFI	0.930
TLI	0.919

Note. SB-χ^2^ = Satorra–Bentler scaled chi-square; RMSEA = root mean square error of approximation; SRMR = standardized root mean square residual; CFI = comparative fit index; TLI = Tucker–Lewis index.

## Data Availability

The data supporting the findings of this study are available from the corresponding author upon reasonable request. The data are not publicly available due to participant privacy and ethical considerations.
